# Synthesis and Characterization of New Functional Photo Cross-Linkable Smart Polymers Containing Vanillin Derivatives

**DOI:** 10.3390/gels2010003

**Published:** 2016-01-14

**Authors:** Momen S.A. Abdelaty, Dirk Kuckling

**Affiliations:** 1 Chemistry Department, University of Paderborn, Warburger Straße 100, D-33098 Paderborn, Germany; 2 Polymer Lap, Chemistry Department, Faculty of Science, Al-Azhar University, Assiut 71524, Egypt; momensayed2007@yahoo.com

**Keywords:** vanillin, *N*-isopropyl acrylamide, photo cross-linking, hydrogel films, temperature responsive, swelling, lower critical solution temperature, surface plasmon resonance

## Abstract

The synthesis of new functional monomers based on vanillin is reported. The monomers further were used in the synthesis of different temperature-responsive photo cross-linkable polymers via free radical polymerization with *N*-isopropyl acrylamide and a maleimide photo cross-linker. These polymers were characterized by NMR, FTIR and UV spectroscopy, as well as gel permeation chromatography (GPC) and differential scanning calorimetry (DSC). Critical solution temperatures were determined by UV spectroscopy. Hydrogel thin films were formed by spin coating of a polymer solution over gold with adhesion promotor followed by cross-linking by UV irradiation. The swelling properties were determined by surface plasmon resonance coupled with optical waveguide spectroscopy. The swelling behavior of the hydrogel films was determined as a function of temperature. The incorporation of a dialkyl amino group compensated the hydrophobic effect of the vanillin monomer. Transition temperatures in the physiological range could be obtained.

## 1. Introduction

Smart polymers or stimuli-responsive polymers have been widely used due to their practical interests, and several types of responsiveness, including temperature, pH, light, pressure, magnetic and electrical fields, have been reported [1]. These kinds of materials were known in nature in some examples, like the leaves of Mimosa pudica and Venus flytrap [2]. These polymers and hydrogels are responsive towards more than one stimulus, but the majority of studies have focused on pH and temperature variation [1,3,4,5,6,7]. This has been achieved by a combination of ionizable and hydrophobic functional groups [8,9,10,11]. Several authors have recently presented their advances in this field [12,13,14,15,16,17,18,19,20,21,22,23,24,25,26]. The lower critical solution temperature (LCST) behavior is associated with a critical temperature (*T*_c_) at which the polymer solution undergoes phase separation from one phase (isotropic state) to two phases (anisotropic state), rich and poor in polymer, respectively [27]. Below *T*_c_, the enthalpy term of the free energy of mixing, related to the hydrogen bonding between the polymer and the water molecules, is responsible for the polymer dissolution. When raising the temperature above *T*_c_, the entropy term (hydrophobic interactions) dominates, leading to polymer precipitation. *T*_c_ of polymers in aqueous solutions can be modulated by incorporating hydrophilic or hydrophobic moieties [27,28,29,30,31]. For example, when *N*-isopropyl acrylamide (NIPAAm) is copolymerized with hydrophilic monomers, such as acryl amide (AAm), the *T*_c_ increases up to about 45 °C when 18 mol % of AAm is incorporated with the polymer; whereas *T*_c_ decreases to about 10 °C when 40 mol % of hydrophobic *N*-tert-butylacrylamide (NtBAAm) is added to the polymer [29].

Functional hydrogels consisting of at least one polymer segment that enables polymer analogous reactions are of increasing importance. Possible routes utilize active ester chemistry, click chemistry, as well as reactions with polymeric anhydrides, epoxides, aldehydes and ketones. Including Michael-type and Friedel–Crafts reactions, as well as methylations, polymer analogous reactions involve almost all high yield organic reactions [32,33,34,35]. The reactive sites allow the modification with a variety of biomolecules for biosensor applications and chromatography based on affinity binding [36,37,38,39,40]; additionally, attaching molecular recognition sites diversified cell culturing, and polymer-assisted drug delivery advanced medication [41]. As mentioned above, responsive behavior can be implemented to create a functional responsive hydrogel or to diversify the responsiveness, *i.e.*, to a magnetic field by incorporating magnetite nanoparticles [42,43]. Recent advances in nanotechnology have increased interest in hydrogel thin films. The advantages of hydrogel thin films have been explored for the fabrication of miniaturized devices with fast response times. Hydrogel thin films have also attracted interest as an approach to responsive surfaces and interfaces, where they compete with grafted polymer layers [44,45]. A 3D polymer network is much more stable at interfaces when compared to polymer brushes, where polymer chains are grafted to the surface via only one functional group, while the polymer network is linked to the surface by multiple anchoring points [2,46]. Surface plasmon resonance spectroscopy coupled with optical waveguide spectroscopy (SPR/OWS) has been widely used in the determination of the LCST behavior of hydrogels [47,48,49,50,51]. Many applications of stimuli-responsive hydrogels have been used in biotechnology, like purification of biomolecules [52,53,54,55,56], switchable wettability [57,58,59,60,61] and sensors (biosensors) [62,63].

Vanillin is a widely-used flavoring, e.g., for food, fragrances and beverages. For sustainability purposes, vanillin can be extracted from natural resources [64] or mass-produced from lignin as a biosource [65,66,67,68]. Vanillin has hydroxyl and aldehyde reactive sites that can be used for chemical modification to produce valuable monomers that can be polymerized into materials with different mechanical and thermal properties [69,70]. Furthermore, vanillin has been converted into vinyl monomers for vinyl ester resins [69,71,72]. By this approach, the resulting vanillin-based monomers possess a reactive aldehyde functionality. Here, we describe the synthesis of different vanillin-based monomers and evaluate their influence on the thermal transition of temperature-responsive polymers. The utilization of the reactive aldehyde group will be reported in a future work.

## 2. Results and Discussion

### 2.1. Synthesis of Functional Monomers and Polymers

**Scheme 1:**
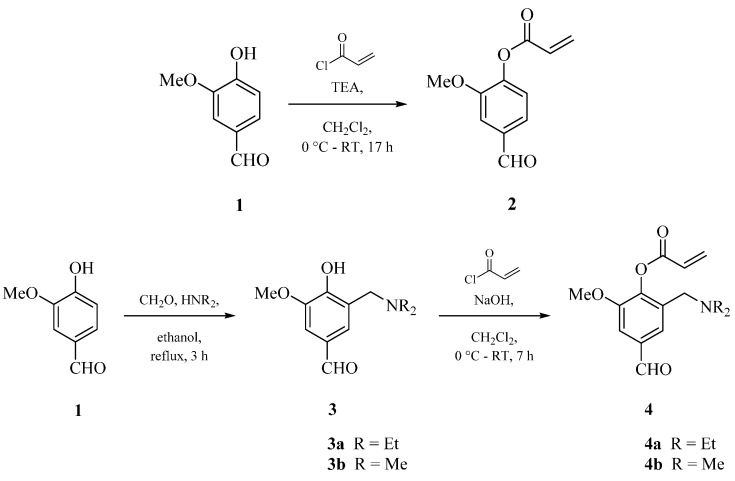
Scheme of the synthesis of vanillin-based monomers (4-formyl-2-methoxyphenylacrylate (VA) **2**, 2-((diethylamino)methyl)-4-formyl-6-methoxyphenyl acrylate (DEAMVA) **4a** and 2-((dimethylamino)methyl)-4-formyl-6-methoxyphenyl acrylate (DMAMVA) **4b**).

The new monomers 4-formyl-2-methoxyphenylacrylate (VA) **2**, 2-((diethylamino)methyl)-4-formyl-6-methoxyphenyl acrylate (DEAMVA) **4a** and 2-((dimethylamino)methyl)-4-formyl-6-methoxyphenyl acrylate (DMAMVA) **4b** were synthesized in one or two steps, as shown in Scheme 1. The first step was the reaction of vanillin with diethylamine for DEAMVA or dimethylamine for DMAMVA and formaldehyde according to a Mannich reaction mechanism. In this reaction, we did not use any catalysis, especially acid catalysis, which are normally used in Mannich reactions. The second step was the reaction with acryloyl chloride in the presence of triethylamine (TEA) or NaOH to form the respective esters. All compounds have been analyzed by ^1^H and ^13^C NMR, as well as IR spectroscopy, and they were in a good agreement with the chemical structures. Details are presented in the experimental part.

**Scheme 2:**
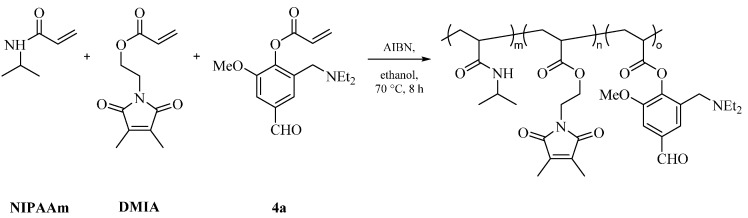
Scheme of the synthesis of the respective photo cross-linkable polymers with vanillin-based monomer DEAMVA. NIPAAm, *N*-isopropyl acrylamide; DMIA, dimethylmaleimidoacrylate.

The free radical polymerization of NIPAAm and VA, DEAMVA or DMAMVA with dimethylmaleimidoacrylate (DMIA) as a photo-reactive moiety was used to synthesize functional temperature-responsive polymers. Different mole ratios of VA, DEAMVA or DMAMVA were used to probe the influence of the relative content. A scheme of the synthesis of the respective photo cross-linkable polymers with vanillin-based monomer DEAMVA is shown in Scheme 2. The polymers are denominated as VA-05-10, whereas VA is the type of vanillin monomer (DE: DEAMVA; DM: DMAMVA); the first number denominates the feed content of the photo cross-linker, and the second number denominates the feed content of the vanillin derivative.

**Figure 1:**
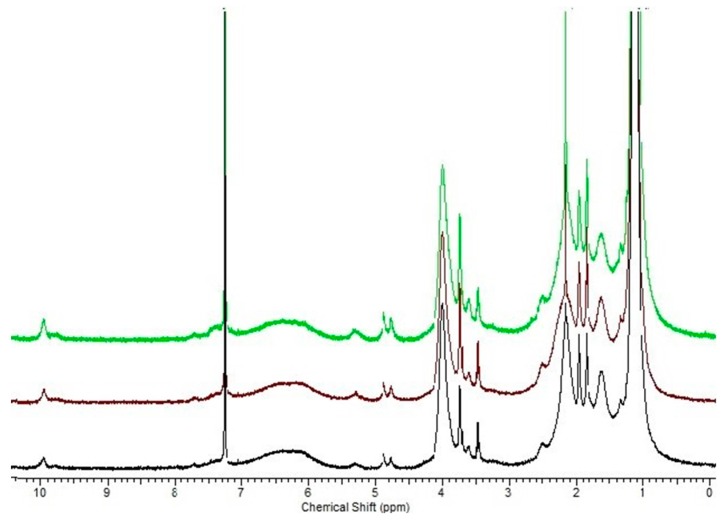
^1^H NMR spectra of DEAMVA (DE)-05-10 (black), DE-05-15 (brown) and DE-05-20 (green).

The ^1^H NMR was performed to determine the actual amounts of DMIA and VA, DEAMVA or DMAMVA in the polymer chain. For this, the integrals of two methyl groups of NIPAAm at 0.76–1.30 ppm, of the two methyl groups of DMIA at 1.89–2.02 ppm and of one hydrogen of the aldehyde group at 9.79–10.10 ppm were used. Representative ^1^H NMR spectra are presented in Figure 1. It can clearly be seen that the aldehyde functional group is still present after copolymerization. In addition, comparable copolymers could be modified with primary amines by imine formation. However, it cannot be excluded that some of the aldehyde functions are destroyed, resulting in relatively low aldehyde content in the polymer. Oppositely, although copolymer parameters are not known, from the comparison of the copolymerization parameters of related structures [73], one can conclude that the phenyl acrylates are less reactive than acrylamides. However, due to the overlap of the signals in the ^1^H NMR spectra, exact calculations were very difficult. The DMIA content was determined with UV spectroscopy, as well. All calculations were in a logic case with the feeding calculation, as shown in Table 1.

**Table 1:** Composition of functional photo cross-linkable smart polymers based on vanillin derivatives VA, DEAMVA and DMAMVA (DM).

Polymer	DMIA (mol %)		2 (mol %)	4a (mol %)	4b (mol %)	Yield (%)
	^1^H NMR	UV				
VA-05-10	5.9	6.0	11.1			83
DE-05-10	4.3	4.2		3.0		60
DE-05-15	4.5	4.7		4.8		57
DE-05-20	4.8	4.8		6.5		58
DM-05-10	4.6	4.7			3.6	66
DM-05-15	4.3	4.4			4.2	56
DM-05-20	4.5	4.5			4.7	43

The number average molecular weight and polydispersity were measured by gel permeation chromatography (GPC). The glass transition temperature *T*_g_, which is important to form a rigid film for photo cross-linking, increased with increasing DEAMVA or DMAMVA composition. The phase transition temperature *T*_c_ was determined for all polymers in water. The phase separation for each polymer was studied by UV-VIS spectroscopy. The results are summarized in Table 2.

**Figure 2:**
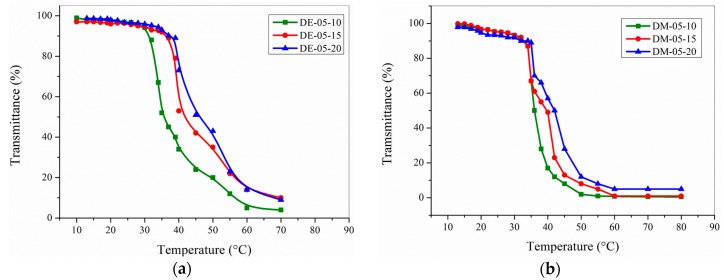
Turbidity measurements of functional thermo-responsive polymers for: (**a**) DEAMVA in water; (**b**) DMAMVA in water (1 wt % of polymer solution).

The *T*_c_ of poly(*N*-isopropyl acrylamide) (PNIPAAm) is readily influenced by hydrophilic or hydrophobic comonomers [11]. Hence, each modification of PNIPAAm might lead to changed *T*_c_ values. Here, the introduction of 11 mol % of the simple vanillin-based monomer **2** led to a decrease of *T*_c_ of about 14 °C compared to the non-modified photo cross-linkable polymer [48]. In order to overcome this shift, vanillin was modified with a methyl amino group having two alkyl groups of different hydrophobicity. The amino group will be slightly charged at weak acid or neutral pH values, which leads to a compensation of the hydrophobic effect. Indeed, the copolymers with DEAMVA and DMAMVA, respectively, showed increased *T*_c_ values in water. No clear difference can be observed for diethyl amino or dimethyl amino modification. The turbidity curve in water showed quite broad transitions, making the determination of *T*_c_ more difficult (Figure 2). This can be attributed to the chemical composition distribution of the polymer chains. Nevertheless, the incorporation of the methyl amino groups increased *T*_c_ by approximately 20 °C. The strong increase might be also due to some additional carboxylic groups resulting from the oxidation of aldehyde groups. Additionally, the copolymers showed quite complex pH behavior. This fact is still under investigation. As a conclusion, these photo cross-linkable polymers should be used at neutral to acidic pH only.

**Table 2:** Characterization of functional photo cross-linkable smart polymers based on vanillin derivatives VA, DEAMVA and DMAMVA.

Polymer	*M*_n_ (g/mol)	*PD*	*T*_g_ (°C)	*T*_c_ (°C)
VA-05-10	20,700	3.1	133.5	13.8
DE-05-10	7600	1.7	131.0	35.0
DE-05-15	5600	1.6	145.0	42.5
DE-05-20	6100	1.5	146.0	46.6
DM-05-10	6500	2.0	128.5	36.0
DM-05-15	5500	1.8	134.0	39.0
DM-05-20	4900	1.9	135.8	35.0

### 2.2. Photo Cross-Linking and Formation of Hydrogel Thin Film

**Figure 3:**
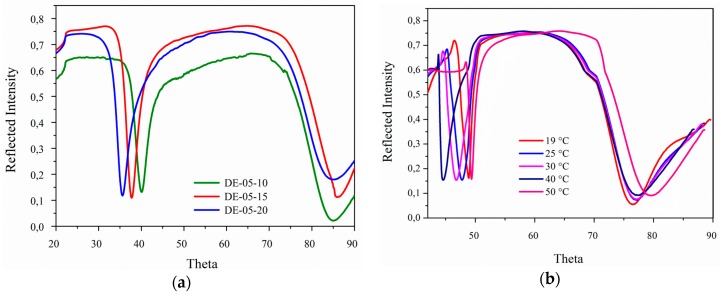
SPR scans of photo cross-linked polymer layers containing DEAMVA: (**a**) in the dry state for different DEAMVA contents; (**b**) in the swollen state for DE-05-15 at different temperatures.

The formation of a hydrogel layer of vanillin-based copolymers was obtained by photo cross-linking through [2 + 2] cyclodimerization of the dimethyl maleimide moieties. In order to investigate the swelling properties by surface plasmon resonance (SPR) spectroscopy, gold-coated LaSFN9 glass with an approximately 50-nm Au film thickness was used as the support. The gold slide was immersed in a solution of 5 mM thioacetic acid 3-(3,4-dDimethyl-2,5-dioxo-2,5-dihydro-pyrrol-yl)-Ppropyl ester (DMITAc) adhesion promoter. Then, the polymer solution was spin coated. It was observed that the used solvent affects the homogeneity of the surface. Solvents like cyclohexanone worked fine, whereas DMSO or DMF failed to form homogeneous films. Films were prepared in a manner such that the dry films were thick enough (approximately 300–500 nm) to show the first waveguide mode (Figure 3a). This is important, since the curve can be fitted to the plasmon minimum, as well as waveguide mode to determine the film thickness, as well as the refractive index of the dry layer. The photo cross-linking has been done in the near UV (300–430 nm). For this reason, the photosensitizer thioxanthone was always used. It transfers the energy of absorbed light to the polymer’s photo-reactive moieties, causing them to enter the excited state and perform the cross-linking reaction. Factors that influenced the photo cross-linking are irradiation wavelength and intensity, reaction time, composition of the photo-reactive polymers and the presence of the photo sensitizer. The presence of the adhesion promoter resulted in surface attachment of the hydrogel to the substrate. The maleimide group in the adhesion promoter reacted with a similar group present in the spin-coated photo cross-linkable polymer through the above-mentioned [2 + 2] cyclodimerization. The Au–S bond, on the other hand, resulted in the coordination covalent bond attachment of the hydrogel to the substrate.

The surface plasmon resonance measurements were carried out according to the Kretschmann configuration. The LaSFN9 glass was optically matched to the base of the glass prism. Monochromatic light from a He/Ne laser at 633 nm was directed through the prism. The external angle of incidence (θ) was varied with a goniometer, and then, the light was collected with a photodiode. The reflected intensity *versus* angle was recorded (Figure 3). The angle-dependent reflectivity was described by the Fresnel equation for a multilayer system [47,48,49]. The base system consisted of LaSFN9 glass, gold, adhesion promoter (DMITAc) and air. In the case of dry polymer, an additional layer for the thin film was added. For the swelling case, the refractive index of deionized water and buffer solution, respectively, was used to model the continuous layer. During swelling and collapse, the refractive index and thickness of the gel layers changed simultaneously. This can be seen by a shift of the plasmon minimum, as well as the waveguide mode still present in the swollen state. Representative spectra are shown in Figure 3b. Waveguide modes can be guided within layers thicker than 200 nm in the dry state and 500 nm in the swollen state. Hence, the combination of SPR and OWS provided an additional feature to determine the layer thickness, as well as the refractive index of the hydrogel simultaneously. From the refractive index, the volume degree of swelling can be estimated [48]. Temperature control was achieved by placing the sample on a hot stage, on which the stage was heated and cooled, resistively, by a Peltier element to adjust the temperature within 15–55 °C.

**Figure 4:**
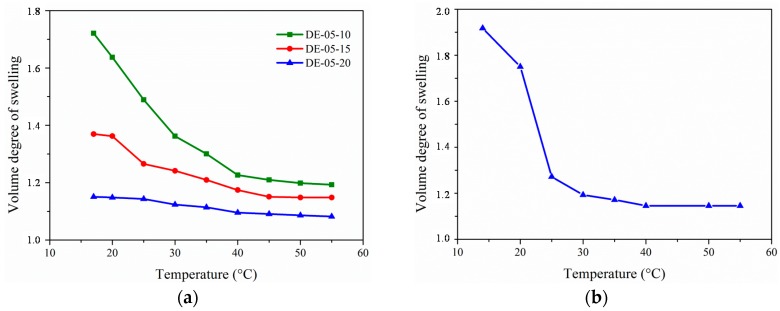
Volume degree of swelling *vs.* temperature of photo cross-linked hydrogels in water; (**a**) DE-05-10, DE-05-15 and DE-05-20; (**b**) DM-05-20.

Figure 4 shows the swelling behavior of different hydrogel layers. A decrease in the volume degree of swelling with rising temperature occurred. As already seen for the soluble polymers, the temperature-induced phase transition was more pronounced for copolymers with vanillin derivatives containing the dimethyl amino groups. With higher contents of DEAMVA in the hydrogel layer, the swelling was decreased. It turned out that the chemical composition distribution caused a broad transition range. This might be due to π–π-stacking of vanillin moieties, resulting in additional dynamic cross-links. Furthermore, carboxylic groups resulting from the accidental decomposition of the aldehyde group can interact with the dialkyl amino groups by Coulomb interactions, again forming additional junction points. With the increase of the DEAMVA content, the possibility of those interactions increases, leading to a decreased swelling. In the case of DMAMVA, the oxidation did not take part to such an extent. Hence, the swelling transition is more pronounced. To prove this hypothesis, a different synthetic approach had to be developed to prevent decomposition of the aldehyde group.

## 3. Conclusions

In this work, new monomers based on vanillin were copolymerized with NIPAAm and DMIA to obtain photo cross-linkable polymers. Surface-attached photo cross-linked thin gel layers were prepared. Their transition temperature *T*_c_ were determined for both soluble polymers and cross-linked gels. The swelling behavior of the thin polymer gel layers was investigated by a combination of surface plasmon resonance (SPR) and optical waveguide spectroscopy (OWS). The incorporation of methyl dialkyl amino functionality could compensate the hydrophobic character of the vanillin backbone with respect to the influence of *T*_c_. Transition temperatures in the physiological range could be obtained. The polymers possess aldehyde functionalities that in the future will be used to bind reversibly-active compounds.

## 4. Experimental Section

### 4.1. Instrumentation

^1^H and ^13^C NMR spectra were recorded on a Bruker AV 500 spectrometer in CDCl_3_ at 500 MHz and 125 MHz, respectively. The solvent signal was used as the internal standard. IR spectra were recorded on the Vertex 70 Fourier transform infrared instrument. The samples were milled with KBr and pressed into pellets.

Molecular weights and polydispersity (PD) were analyzed employing size exclusion chromatography (SEC). As eluent, chloroform (containing 0.1 vol % triethylamine) with a flow rate of 0.75 mL/min (Jasco 880-PU pump) was used with a RI-Detector (Waters, Milford, MA, USA) and toluene as the internal standard at 30 °C. The samples (15 mg/mL) were injected by hand via a 20-μL loop. PSS-SDV columns (Polymer Standards Service, Germany) filled with 5-μm gel particles with a defined porosity of 10^6^ Å (guard), 10^5^ Å, 10^3^ Å and 10^2^ Å, respectively, were used. Molecular weight determination was based on narrow polystyrene standards. The Differential Scanning Calorimeter Pyris 1 (Perkin Elmer, Waltham, MA, USA) was used for the determination of *T*_g_ of solid polymers. The thermogram was recorded at a heating and cooling rate of 5 °C/min. UV-VIS spectra were recorded on a Lambda 45 Spectrometer (Perkin Elmer). For the *T*_c_ determination, a polymer solution of 1 wt % in water was used. As *T*_c_, the value at 50% transmission was used. The spin coater model G3P-8 SpinCoat (Speciality Coating System, Inc., Amherst, NH, USA) was used to prepare thin polymer films on the substrates with 250 rpm for 30 s and 1000 rpm for 140 s (polymer concentration 10 wt %). A UV Hg-lamp (OSRAM, Munich, Germany, 100 W) equipped with an optical lens and a mirror was used for the photo cross-linking process. An SPR along with OWS setup (Restech, Munich, Germany) was used to determine the film thickness and swelling ratios of photo cross-linked thin films. A He–Ne laser beam with a wavelength of 632.8 nm was used for excitation of SPs in the Kretschmann configuration. The substrate was LaSFN9 glass slides with an approximately 50-nm gold film. The Au film was deposited by PVD (physical vapor deposition, tectra GmbH, Frankfurt am Main, Germany). After adsorption of the DMITAc adhesion promoter on Au, photo cross-linkable polymer solution was spin coated and irradiated with UV light. For measuring temperature-dependent swelling, 3–4 mL of distilled water were injected manually into the SPR cell (the SPR cell consists of a sample holder, LaSFN9 glass with gold coating, a prism and a Peltier element to maintain the temperature). The temperature inside the cell was measured with a thermocouple of 0.1 °C accuracy.

### 4.2. Materials

*N*-isopropyl acrylamide (NIPAAm; Acrōs, Geel, Belgium) was recrystallized from distilled hexane. Vanillin (99% Acrōs), dimethylamine and diethylamine (Merck, Darmstadt, Germany), triethylamine (Merck), 2-hydroxyethylamine (Acrōs), dimethyl maleic anhydride (98%, Aldrich, St. Louis, USA) and acryloyl chloride (AcrCl, Merck) were used as received. 2,2′-Azobis(isobutyronitrile) (AIBN) was recrystallized from methanol. Dioxane, tetrahydrofuran (THF) and diethyl ether were distilled over potassium hydroxide.

#### 4.2.1. Synthesis of the Dimethylmaleimidoacrylate Photo Cross-Linker

The dimethylmaleimidoacrylate (DMIA) monomer was prepared according to the literature [74].

#### 4.2.2. Synthesis of Thioacetic Acid 3-(3,4-Dimethyl-2,5-dioxo-2,5-dihydro-pyrrol-yl)-Propyl Ester Adhesion Promoter

The DMITAc was prepared according to the literature [47].

#### 4.2.3. Synthesis of 4-Formyl-2-Methoxyphenylacrylate

In a two neck flask fitted with an argon balloon, vanillin (4-hydroxy-3-methoxy benzaldehyde) (8 g, 0.052 mol) was dissolved in dry CH_2_Cl_2_ (100 mL). Under vigorous stirring, TEA (10.52 g, 0.1 mol) was added. The reaction mixture was cooled in an ice bath to 0–5 °C. Acryloyl chloride (5.4 g, 0.059 mol) was added drop wise. The yellowish suspension was stirred at 5 °C for 1 h, then allowed to stir at RT overnight. The precipitate was filtered, and the solvent was evaporated under reduced pressure. The product was extracted by CH_2_Cl_2_ and washed three times with distilled water, one time with sodium carbonate and one time with 0.1 M HCl. The organic phase was dried with MgSO_4_ overnight, then filtered, and the product was distilled using an oil pump; yield 85%; the colorless oil changed to a white, pasty solid after cooling overnight in a refrigerator.

^1^H NMR (500 MHz, CDCl_3_): δ (ppm) = 3.73 (s, 3 H, OCH_3_), 5.92 (dd, *^2^J* = 0.7 Hz, *^3^J* = 10.5 Hz, 1 H, =CH_2_), 6.23 (dd, *^3^J* = 10.5 Hz, *^3^J* = 17.3 Hz, 1 H, =CH), 6.49 (dd, *^2^J* =0.7 Hz, *^3^J* = 17.3 Hz, 1 H, =CH_2_), 7.12 (d, *^3^J* = 8.0 Hz, 1 H, 6-Ar–CH), 7.34 (dd, *^3^J* = 8.0 Hz, *^4^J* = 1.5 Hz, 1 H, 5-Ar–CH), 7.37 (d, *^4^J* = 1.3 Hz, 1 H, 3-Ar–CH), 9.96 (s, 1 H, CHO).

^13^C NMR (125 MHz, CDCl_3_): δ (ppm) = 55.91 (1 C, OCH_3_), 111.09 (1 C, 3-Ar–CH), 123.36 (1 C, 5-Ar–CH), 124.24 (1 C, 6-Ar–CH), 127.08 (1 C, =CH), 133.16 (1 C, =CH_2_), 135.26 (1 C, 4-Ar–C), 144.62 (1 C, 1-Ar–CH), 151.09 (1 C, 2-Ar–C), 163.22 (1 C, COO), 190.99 (1 C, CHO).

IR (KBr): *ν* (cm^−1^) = 2970–2950 (s) (CH_2_, CH_3_), 2840 (m) (OCH_3_), 1745 (s) (COO), 1695 (s) (CHO), 1600 (s) (C=C), 885–784 (m) (Ar–CH).

#### 4.2.4. Synthesis of 2-((Diethylamino)methyl)-4-Formyl-6-Methoxyphenyl Acrylate (**2**)

Step 1: Synthesis of 3-((diethylamino)methyl)-4-hydroxy-5-methoxy-benzaldehyde (**3a**).

In a 250-mL round-bottomed flask fitted with a reflux condenser, vanillin (4-hydroxy-3-methoxy benzaldehyde) (10.0 g, 0.065 mol), formaldehyde (10.0 g, 0.33 mol) and diethylamine (10.0 g, 0.136 mol) were dissolved in ethanol (150 mL). The mixture was refluxed for 3 h. Then, the mixture was allowed to cool to room temperature. The solvent was removed under reduced pressure to collect the product; yield 97%; yellowish white solid.

^1^H NMR (500 MHz, CDCl_3_): δ (ppm) *=* 1.18 (t, *^3^J* = 7.2 Hz, 6 H, CH_3_), 2.73 (q, *^3^J* = 7.2 Hz, 4 H, CH_2_CH_3_), 3.92 (s, 2H, NCH_2_), 3.94 (s, 3 H, OCH_3_), 7.25 (br, 1 H, 2- or 6-Ar–CH), 7.34 (d, *^4^J* = 1.6 Hz, 1 H, 2- or 6-Ar–CH), 9.78 (s, 1 H, CHO).

^13^C NMR (125 MHz, CDCl_3_): δ (ppm) = 10.82 (2 C, CH_3_), 46.35 (2 C, CH_2_CH_3_), 55.85 (1 C, NCH_2_), 56.01 (1 C, OCH_3_),109.68 (1 C, 6-Ar–CH), 120.84 (1 C, 2-Ar–CH), 125.75 (1 C, 3-Ar–C), 127.99 (1 C, 1-Ar–C), 148.65 (1 C, 4-Ar–C), 154.87 (1 C, 5-Ar–C), 191.65 (1 C, CHO).

IR (KBr): *ν* (cm^−1^) = 2987 (s) (CH_2_, CH_3_), 1706 (s) (C=O), 1650 (s) (C=C), 868–820 (m) (Ar–CH).

Step 2: Synthesis of 2-((diethylamino)methyl)-4-formyl-6-methoxyphenyl acrylate (**4a**) (DEMAVA).

In a two-neck flask fitted with an argon balloon, **1** (13.9 g, 0.058 mol) was dissolved in dry CH_2_Cl_2_ (200 mL) and stirred strongly, and sodium hydroxide (10.0 g, 0.25 mol) was added. The reaction mixture was allowed to cool in an ice bath to 0–5 °C. Then acryloyl chloride (5.4 g, 0.059 mol) was added drop wise. The yellowish suspension was stirred at 5 °C for 1 h and then allowed stirred at RT for 6 h. The precipitate was filtered, and the solvent was evaporated under reduced pressure. The product was dissolved in CH_2_Cl_2_ and washed three times with DI water, one time with 0.1 M Na_2_CO_3_ and again with DI water. After drying over MgSO_4_ overnight, the solvent was removed under reduced pressure to collect the product; yield 75%; orange viscous liquid.

^1^H NMR (500 MHz, CDCl_3_): δ (ppm) *=* 1.01 (t, *^3^J* = 7.1 Hz, 6 H, CH_3_), 2.50 (q, *^3^J* = 7.1 Hz, 4 H, CH_2_CH_3_), 3,52 (s, 2 H, NCH_2_), 3.87 (s, 3 H, OCH_3_), 6.05 (dd, *^2^J* = 1.2 Hz, *^3^J* = 10.5 Hz, 1 H, =CH_2_), 6.36 (dd, *^3^J* = 10.5 Hz, *^3^J* = 17.3 Hz, 1 H, =CH), 6.64 (dd, *^2^J* = 1.2 Hz, *^3^J* = 17.3 Hz, 1 H, =CH_2_), 7.39 (d, *^4^J* = 1.7 Hz, 1 H, 3- or 5-Ar–CH), 7.66 (d, *^4^J* = 1.7 Hz, 1 H, 3- or 5-Ar–CH), 9.95 (s,1H, CHO).

^13^C NMR (125 MHz, CDCl_3_): δ (ppm) *=* 11.64 (2 C, CH_3_), 47.00 (2 C, CH_2_CH_3_), 51.58 (1 C, NCH_2_), 56.20 (1 C, OCH_3_), 108.60 (1 C, 5-Ar–CH), 126.45 (1 C, 3-Ar–CH), 127.19 (1 C, =CH), 127.19 (1 C, 2-Ar–C), 130.52 (1 C, 4-Ar–C), 134.52 (1 C, =CH_2_), 143.49 (1 C, 1-Ar–C), 152.11 (1 C, 6-Ar–C), 163.03 (1 C, COO), 191.52 (1 C, CHO).

IR (KBr): *ν* (cm^−1^) = 2915, 2834 (s) (CH_2_, CH_3_), 1639 (s) (C=O), 1600 (s) (C=C), 866–820 (m) (Ar–CH).

#### 4.2.5. Synthesis of 2-((Dimethylamino)methyl)-4-Formyl-6-Methoxyphenyl Acrylate (**4**)

Step 1: Synthesis of 3-((dimethylamino)methyl)-4-hydroxy-5-methoxy-benzaldehyde (**3b**).

In this experiment, dimethylamine was used. The same conditions were used as discussed earlier for DEAMVA Step 1.

^1^H NMR (500 MHz, CDCl_3_): δ (ppm) *=* 2.37 (s, 6 H, NCH_3_), 3.75 (s, 2 H, NCH_2_), 3.93 (s, 3 H, OCH_3_), 6.37 (br, s, 1 H, OH), 7.15 (d, 1 H, *^4^J* = 1.8 Hz, 2- or 6-Ar–CH), 7.33 (d, 1 H, *^4^J* = 1.8 Hz, 2- or 6-Ar–CH), 9.76 (s, 1 H, CHO).

^13^C NMR (125 MHz, CDCl_3_): δ (ppm) *=* 44.32 (2 C, NCH_3_), 56.01 (1 C, NCH_2_), 62.21 (1 C, OCH_3_), 109.97 (1 C, 6-Ar–CH), 123.70 (1 C, 2-Ar–CH), 125.21 (1 C, 3-Ar–C), 128.09 (1 C, 1-Ar–C), 148.68 (1 C, 4-Ar–C), 154.54 (1 C,5-Ar–C), 190.67 (1 C, CHO).

IR (KBr): *ν* (cm^−1^) *=* 2977 (s) (CH_2_, CH_3_), 1742 (s) (C=O), 1646 (s) (C=C), 865–818 (m) (Ar–CH).

Step 2: Synthesis of 2-((dimethylamino)methyl)-4-formyl-6-methoxyphenyl acrylate (**4b**) (DMAMVA).

The same conditions were used as discussed earlier for DEAMVA Step 2; yield 73%; orange viscous liquid.

^1^H NMR (500 MHz, CDCl_3_): δ (ppm) *=* 2.19 (s, 6 H, CH_3_), 3.37 (s, 2 H, NCH_2_), 3.84 (s, 3 H, OCH_3_), 6.03 (dd, *^2^J* = 1.1 Hz, *^3^J* = 10.5 Hz, 1 H, =CH_2_), 6.34 (dd, *^3^J* = 10.5 Hz, *^3^J* = 17.3 Hz, 1 H, =CH), 6.64 (dd, *^2^J* = 1.1 Hz, *^3^J* = 17.3 Hz, =CH_2_), 7.37 (d, 1 H, *^4^J* = 1.7 Hz, 3- or 5-Ar–CH, 7.34 (d, 1 H, *^4^J* = 1.7 Hz, 3- or 5-Ar–CH), 9.91 (s,1 H, CHO).

^13^C NMR (125 MHz, CDCl_3_): δ (ppm) *=* 45.42 (2 C, CH_3_), 55.72 (1 C, NCH_2_), 62.41 (1 C, OCH_3_), 108.75 (1 C, 5-Ar–CH), 122.37 (1 C, 3-Ar–CH), 126.25 (1 C, =CH), 127.30 (1 C, 2-Ar–C), 132.22 (1 C, 4-Ar–C), 134.39 (1 C, =CH_2_), 143.50 (1 C, 1-Ar–C), 152.51 (1 C, 6-Ar–C), 162.81 (1 C, COO), 191.64 (1 C, CHO).

IR (KBr): *ν* (cm^−1^) = 2935 (s) (CH_2_, CH_3_), 1665 (s) (C=O), 1610 (s) (C=C), 873–826 (m) (Ar–CH).

#### 4.2.6. Synthesis of Photo Cross-Linkable Poly(NIPAAm-*co*-DEAMVA-*co*-DMIA) (**5**) and Poly(NIPAAm-*co*-DMAMVA-*co*-DMIA) (**6**)

General procedure: In a 100-mL round-bottomed flask DMIA (5 mol %) was added 2-((diethylamino)methyl)-4-formyl-6-methoxyphenyl acrylate or 2-((dimethylamino)methyl)-4-formyl-6-methoxyphenyl acrylate, respectively (10, 15 and 20 mol %) and NIPAAm (2.00 g, 17.6 mmol) in ethanol (50 mL), and AIBN (ca. 10 mg) was also added. The reaction mixture was purged in argon for 20 min and then heated in an oil bath to 70 °C for 8 h. After cooling to room temperature, the polymer was precipitated in diethyl ether at −50 °C. The precipitate was collected, redissolved in THF and reprecipitated in diethyl ether to remove the unreacted monomers and impurities. Polymerization has also been done in 1,4-dioxane.
